# Nomophobia and other psychological symptoms among nursing students community

**DOI:** 10.1192/j.eurpsy.2025.833

**Published:** 2025-08-26

**Authors:** M. Efstathiou, A. Grammeniati, K. M. Panoutsopoulou, V. Kakaidi, S. Mantzoukas, M. Gouva, E. Dragioti

**Affiliations:** 1 Department of Nursing, Research Laboratory Psychology of Patients, Families & Health Professionals, School of Health Sciences, University of Ioannina; 2 Department of Nursing, Research Laboratory Integrated Care, Health & Well-being, School of Health Sciences, University of Ioannina, Ioannina, Greece

## Abstract

**Introduction:**

Nomophobia among university students is recognized as an addictive issue, as their attention is often difficult to divert from smartphones, especially during class. This issue is increasingly evident among nursing students, who frequently check their smartphones during class (p < 0.001), making nomophobia an important concern.

**Objectives:**

We conducted an umbrella review aimed at assessing the prevalence of different psychological and behavioral symptoms among nursing students, including nomophobia, anxiety, sleep disturbances, fear, and stress.

**Methods:**

This meta-synthesis combined evidence from 20 systematic reviews and meta-analyses, incorporating 354 primary studies. Publication records were retrieved from PubMed, CINAHL, PsycINFO, and Scopus. The methodological quality of each meta-analysis was assessed using the AMSTAR-2 tool. Reporting followed the PRISMA guideline checklist.

**Results:**

Our synthesis revealed that 28% (95% CI: 24%–33%) of nursing students experience psychological and behavioral symptoms. Nomophobia/smartphone addiction was observed at 30% (95% CI: 12%–49%). Other prevalent symptoms included anxiety at 29% (95% CI: 17%–40%), sleep disturbances at 48% (95% CI: 5%–91%), stress at 27% (95% CI: 17%–37%), and fear at 41% (95% CI: 7%–75%).

**Conclusions:**

Our findings suggest that nursing students are increasingly involved in nomophobia. As smartphones play a central role in daily life, digital detoxification is not easy. Although our research did not explore the relationship between nomophobia and other symptoms, the presence of issues such as anxiety, sleep disturbances, fear, and stress in nursing students warrants further investigation.

**Disclosure of Interest:**

None Declared

